# The Hybrid Subischial Socket for Persons With Transfemoral Amputation: Gait Parameters and Clinical Assessment of a Case Series

**DOI:** 10.33137/cpoj.v4i1.36252

**Published:** 2021-07-14

**Authors:** R Pellegrini, G Denza, S Brunelli, D Zenardi, M Imperio, G Vannozzi, M Traballesi

**Affiliations:** 1 ITOP Spa, Officine Ortopediche, Palestrina, Rome, Italy.; 2 Fondazione Santa Lucia, Scientific Institute for Research, Hospitalization and Health Care, Rome, Italy.; 3 Department of Movement, Human and Health Sciences, Foro Italico, University of Rome, Rome, Italy.

**Keywords:** Artificial Limb, Prosthetic Socket, Gait Analysis, Transfemoral Amputation, Locomotion, Ischial Containment, Socket Comfort

## Abstract

**BACKGROUND:**

The subischial socket interface design is a promising new shape of socket for persons with transfemoral amputation. Typically, the proximal trim line is located distal to the ischial tuberosity, improving comfort in prosthetic users without interfering with gait parameters compared to Ischial Containment Socket (ICS). No studies have investigated the performances of a subischial sockets with suction suspension system. A new subischial socket (Hybrid Subischial Socket - HySS) combined with a hypobaric passive suspension system has been recently developed.

**OBJECTIVE:**

To assess the effects of HySS in terms of comfort, hip range-of-motion and gait parameters.

**METHODOLOGY:**

Three persons with transfemoral amputation were tested first using their usual ICS and then after one month of continuous use of HySS.

**FINDINGS:**

The following parameters improved in all participants using HySS: 1) hip range-of-motion, 2) walking speed and distance, 3) Timed-Up-and-Go-Test time, 4) stride length, 5) double support duration, 6) peak value of hip extension during stance, 7) satisfaction with the prosthesis.

**CONCLUSION:**

These findings suggest that the use of HySS could allow improvements for prosthetic use.

## INTRODUCTION

Current trends relating to the design of the prosthesis for persons with transfemoral (TF) amputation lead to sockets and suspension systems that allow for a hip range of motion that is as close as possible to the physiological one, and that do not interfere with muscle activity. A socket with these characteristics should improve walking of persons with TF amputation.^1,2^

The socket is the interface between the prosthesis and the appendicular skeleton via residual limb soft tissue. The socket shape aims to ensure a comfortable use of the prosthesis both in static and in dynamic phases without causing pain.^3^ Despite the improvement in technology in recent years, about 20% of persons with TF amputation are reported not walking at all at home while about 50% do not use the prosthesis outside.^4^

The most common transfemoral sockets is the Ischial Containment Socket (ICS).^5^ One drawback is the limitation of hip motion, in particular the hip extension, because it encloses the ischial tuberosity and the ramus within the socket. This shape is also reported to cause discomfort when the user is sitting.^6^

The Marlo Anatomical Socket (MAS) is an evolution of the ICS. MAS users have shown a significant improvement of gait efficiency and prosthesis-related perceived mobility compared to ICS. Although the ischium and gluteus maximum are not included in the MAS because of the lowered posterior shelf, even requires an interaction with the pelvis due to lateral containment.^2^

The subischial socket has been a recent development in socket design.^7^ In this socket the proximal trim line is located distal to the ischial tuberosity and it does not interact with the pelvis.

**Figure 1:**
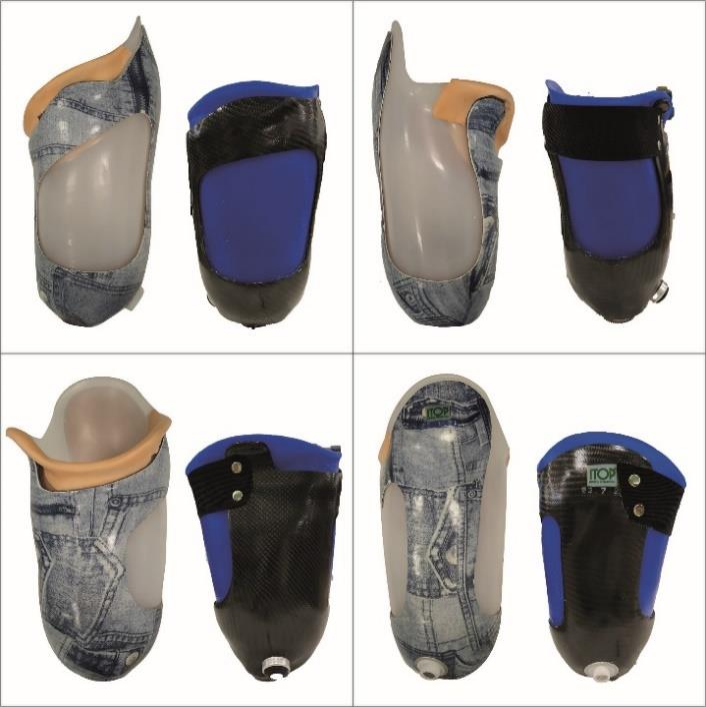
Differences between Ischial Containment Socket (left side - grey socket) and Hybrid Subischial Socket (right side - blue socket). Top left: frontal view. Top right: posterior view. Bottom left: medial view: Bottom right: lateral view.

In the last decade some studies had shown how the subischial socket could improve the spatiotemporal gait parameters, the functional performance with the prosthesis and the comfort compared to the ICS.^2,8–11^ The vacuum assisted suspension system was used in all these studies. More recently the Northwestern University, Prosthetic Orthotic Center, Chicago, Illinois, USA has described the construction technique necessary to create a subischial socket with a suction suspension system: the NU-FLEX SIS.^12^ This technical note showed that suspension systems can involve differences in socket shape compared to the subischial socket ensured by vacuum. A subischial socket (Hybrid Subischial Socket - HySS) has been developed, embedding a suspension system that is ensured by a hypobaric liner Seal-In X^®^ without an external sleeve and without vacuum. Its internal shape has four areas of tissue compression obtained with a casting technique and without rectification procedures as previously described in the literature.^7,12^ The HySS includes an inner socket made of biomedical silicone and an external carbon fiber frame. Figure 1 shows the differences between ICS and HySS.

**Figure 2:**
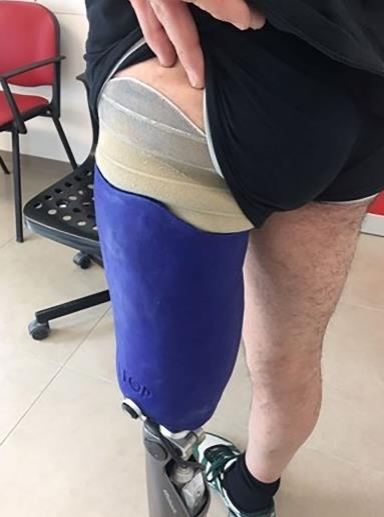
Posterior view of HySS. In this case, the socket is coated with silicone for aesthetic purposes.

The shape and the principles of manufacturing of HySS, as in the other subischial sockets, may represent a benefit for the ICS prosthesis users. The pelvis is free from the contact with the socket, thus an increase in hip range of motion (RoM) should be observed (Figure 2).^13^

An increase in RoM should “free” the hip during walking with better performances in terms of speed, endurance, motor ability and comfort during prosthesis use.

To the best of our knowledge, no previous studies have investigated the functional effects of a subischial socket with suction suspension system. This report describes the differences in terms of gait parameters, hip RoM and satisfaction with the prosthesis following the change of the socket (from ICS to HySS) in three persons affected by TF amputation.

## METHODOLOGY

### 2.1 Participants

We randomly selected the persons with TF amputation among those accessing the local Prosthetics-Orthotics Center, informing them about the opportunity to try the new socket. The inclusion criteria included being over the age of 18, being able to provide informed consent, having a transfemoral amputation, and being a prosthesis user. Informed consent was obtained from the participants after they were provided with an accurate description of the HySS and the purpose of the tests they would be subjected to. This case study was a pilot study; a forthcoming larger study is planned for which ethics committee approval is pending.

### 2.2 Testing protocols

Gait kinematic was measured using a SMART DX700 from BTS Bioengineering (Milan, Italy), which consisted of eight infrared cameras, used to record the position of 22 passive markers, applied on the subjects following the Davis protocol,^14^ at a rate of 250 Hz. Kinematics data were then combined with the kinetic one obtained using four force plates (BTS P-6000), each one containing four load cells that use strain gauges.^15^ A recording is also done with two optical cameras BTS Vixta (sampling rate: 25 Hz) to combine each test with a real-time video recording. Data analysis was performed using BTS SMART software, which is associated to the used devices; it calculates spatiotemporal parameters, joint kinematics and kinetics from the raw data acquired by the cameras and the force plates. The covered distance during Six-Minute Walking Test (6MWT) and duration of the Timed-Up-and-Go-Test (TUG) were obtained using one inertial measurement unit (G-Sensor, BTS), firmly attached on the pelvis of the participant^16^ and the relevant gait parameters calculated.^17,18^ For analysis subset of them, the Degree of Asymmetry (DoA) was calculated as in previous studies,^9,19^ because it allows for the assessment of the differences between the contralateral and prosthetic leg during locomotion tasks. In a healthy individual, the asymmetry is lower compared to a person with unilateral lower limb amputation, thus an effective prosthesis should lead to DoA for all measured values as low as possible. The DoA represents the variation between the sound leg (S) and the prosthetic leg (P) which is obtained using the following formula:


$$DoA=\left(\frac{S-P}{S+P}\right)*100$$

#### 2.2.1 Hip range of motion

**Figure 3:**
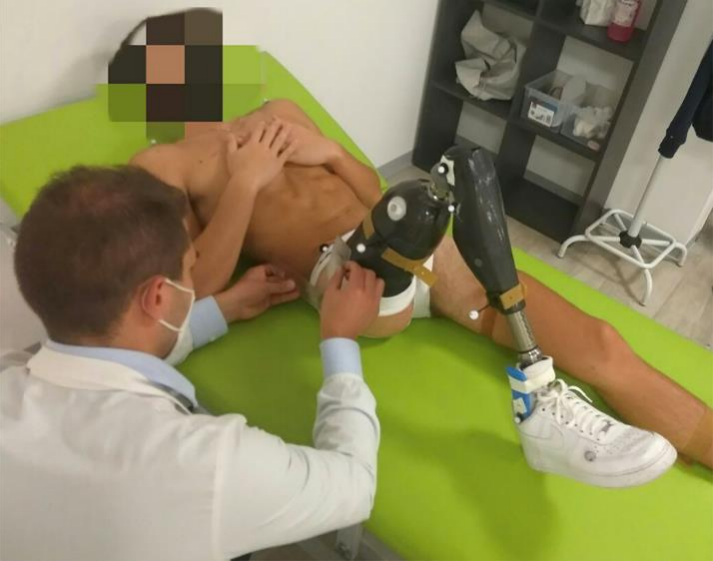
Measurement of hip flexion of the prosthetic side.

Maximum values of hip extension, flexion and abduction on the prosthetic side were measured using a long arm goniometer and the protocol proposed by Norkin.^20^ Four measurements were performed for each variable and, then, the relevant mean values were calculated (Figure 3).

#### 2.2.2 Gait analysis

Subjects were asked to walk on a straight line at a self-selected speed inside the measurement area defined by the cameras. Five trials were recorded, during which at least one gait cycle could be identified using the force plate data. Focusing on a full gait cycle for each leg, based on both kinematics and kinetics measurements and the identification of the heel strike and toe-off events, the mean value of the following parameters was calculated:^21,22^

1) Walking speed (m/s),
2) Cadence (steps/min),
3) Step width (m),
4) Stride length (m),
5) Double support (%),
6) Hip extension (degrees).^23^

For a subset of the above mentioned gait analysis parameters, the DoA was also calculated:^9^

1) Step Length (DoA),
2) Stance Duration (DoA),
3) Swing Duration (DoA),
4) Single Support Duration (DoA).

Hip angles on the sagittal plane of both legs were exported in MATLAB. An algorithm was implemented to identify the peak value of the extension of the prosthetic hip and sound leg during the stance phase, as in a study by Tranberg et al.^13^

#### 2.2.3 Performance tests using a wearable sensor

With an inertial sensor placed on the L5 vertebra, each subject walked on a straight line back and forth for six minutes.^24^ The path was 10 meters long inside the laboratory. The proprietary software (G-Studio) provided the total distance traveled (6MWT).

With the same set-up, the participants were asked to stand up from a chair, to walk for three meters and, then, to turn back and sit down again, recording the amount of time necessary for the patient to perform the task (TUG).^25^

#### 2.2.4 Self-evaluation test

To determine comfort and improvement of quality of life provided by the use of the prosthesis, the participants completed the SATPRO questionnaire which involves 15 items related to the use of the prosthesis in daily life measured using a four-level scale (score 0-45, where 45 means maximum satisfaction).^26^

### 2.3 Timing and socket change

All the mentioned evaluation tools were administered twice. The first measurement occurred after the first evaluation session performed with the ICS socket. Participants were also fit with the HySS at this first appointment. After one month of acclimation to the new socket, participants performed the second evaluation session while wearing the HySS socket. Only the sockets were changed between sessions. Socket alignment was performed by a team of certified experienced prosthetists.

## RESULTS

In this preliminary study, 3 males with TF amputation were enrolled. All of them were K-level 4 prosthetic users, fitted with an ICS socket and a Seal-In suspension system. Proper fit and function of the existing prosthesis were confirmed by a certified prosthetist. The demographic and prosthetic information is reported in Table 1.

**Table 1:** Demographic and prosthetic information of the sample. BMI= Body Mass Index.

Patient	Age (yrs.)	BMI (kg/m^2^)	Side	K-Level	Cause of amputation	Time since amputation	Suspension System	Knee	Foot
1	18	19.58	Right	4	Cancer	10 months	Seal-In	Genium	Pro-Flex
2	35	21.26	Left	4	Trauma	18 years	Seal-In	Genium	Vari-Flex
3	32	26.69	Left	4	Trauma	7 months	Seal-In	Genium	Pro-Flex

As shown in Table 2, no difference was found in terms of variation of cadence, step width and all DoAs in each participant while wearing the ICS or HySS socket. An improvement was observed in the following parameters in all participants donning HySS: 1) passive hip RoM, 2) the distance covered during 6MWT, 3) the time of TUG and the following kinematic and temporal parameters related to gait, 4) stride length, 5) double support duration, 6) walking speed and 7) peak value of hip extension during stance phase for both legs (Table 2).

**Table 2:** Results obtained for each participant with both sockets. The difference (DIF%) is expressed as percentage variation. ICS: Ischial Containment Socket. HySS: Hybrid Subischial Socket. DoA: Degree of Asymmetry.

Measure	Patient 1			Patient 2			Patient 3		
	ICS	HySS	DIF%	ICS	HySS	DIF%	ICS	HySS	DIF%
Hip Range of Motion									
Abduction°	30 ± 6	45 ± 6	+50	18 ± 6	32 ± 5	+77.78	34 ± 4	40 ± 4	+ 17.65
Flexion°	88 ± 11	96 ± 11	+9.09	65 ± 10	95 ± 13	+46.15	77 ± 12	110 ± 12	+42.86
Extension°	21 ± 7	25 ± 6	+ 19.05	10 ± 7	21 ± 9	+ 110	10 ± 8	20 ± 8	+ 100
Gait Analysis (global temporal parameters)									
Walking Speed (m/s)	1.1 ± 0.0	1.2 ± 0.0	+9.09	0.9 ± 0.1	1.1 ± 0	+22.22	1.1 ± 0	1.2 ± 0	+9.09
Cadence (steps/min)	101.8 ± 1.7	104.8 ± 2.6	+2.95	97 ± 3	100.2 ± 1.1	+3.3	108.7 ± 0.8	109.9 ± 2.6	+ 1.1
Gait Analysis (global spatial parameters)									
Step Width (m)	0.10 ± 0.01	0.11 ± 0.01	+ 10	0.12 ± 0.01	0.10 ± 0.01	-16.67	0.17 ± 0.01	0.16 ± 0.03	-5.88
Gait Analysis (P-Leg temporal and spatial parameters)									
Stride Length (m)	1.29 ± 0.04	1.42 ± 0.6	+ 10.0	1.11 ± 0.03	1.29 ± 0.03	+ 16.2	1.21 ± 0.01	1.31 ± 0.03	+8.2
Double Support Duration (%)	15.1 ± 0.8	13.2 ± 0.9	-12.5	17.9 ± 1.3	11.4 ± 0.8	-36.3	19.5 ± 0.4	14.2 ± 1.1	-27.1
Gait Analysis (peak angles during stance phase)									
P-Leg Hip Extension°	-19.9 ± 0.8	-19.7 ± 1.7	-1.0	-3.5 ± 0.9	-8.2 ± 0.6	-134.2	3.4 ± 1.1	1.2 ± 0.9	-64.7
S-Leg Hip Extension°	-14.2 ± 1.7	-14.7 ± 2.2	-3.4	-6.3 ± 1.2	-8.1 ± 0.9	-28.5	1.5 ± 1.9	-4 ± 3	-366.6
Gait Analysis (asymmetry between legs)									
Step Length (DoA)	4 ± 3	-3.9 ± 1.9	-8.18	-1 ± 6	0.1 ± 0.8	1.14	-0.7 ± 0.5	0.9 ± 1.2	1,66
Stance Duration (DoA)	6.2 ± 1.7	6.3 ± 1.4	0.09	5.3 ± 2.2	3.2 ± 1.9	-2.20	1.7 ± 0.9	4.5 ± 2.8	2,85
Swing Duration (DoA)	-10.9 ± 2.8	-10.8 ± 2.9	0,16	-10 ± 5	-5 ± 3	5.31	-3.9 ± 1.8	-8 ± 5	-4.75
Single Support Duration (DoA)	8 ± 6	11 ± 3	2,35	9 ± 6	5 ± 6	-3.99	1.0 ± 2.5	8 ± 7	7.20
Timed-Up and Go									
Duration (s)	15.2 ± 0.5	13.1 ± 1.5	-13.82	15.3 ± 0.7	13.6 ± 0.8	-11.11	10.7 ± 0.7	9.1 ± 0.3	-14.95
6-Minute Walking Test									
Distance (m)	300	340	+ 13.33	NA	320		270	380	+40.74
SAT-PRO									
Score	43	45	+4.6	35	43	+22.8	28	34	+21.4

Based on self-evaluation tests that are known to be indicative of the participants’ opinion of the sockets, all participants reported greater satisfaction with HySS (Table 2)

## DISCUSSION

All three participants in this case series demonstrated improvement in hip RoM, some gait parameters, and satisfaction with the prosthesis when using the HySS compared to the ICS.

The increase in passive hip RoM confirms the sub-ischial design overcomes one disadvantage of the ischial containment socket, which is characterized by an increased constraint on the hip of the affected limb, as shown by other authors.^2,8,26^ Our results clearly indicate increased RoM in both ab/adduction and flexion-extension of the hip, obtaining higher maximum angles, with percentage change that reaches +110% in hip extension for one patient. Greater degree of hip flexion is also achieved during walking and for both the prosthetic and the sound limbs, with values obtained for all patients that are closer to those observed in normal gait.

The SATPRO results suggest an increased overall comfort using the HySS, probably due to the greater RoM. Two patients reported a remarkable improvement in SATPRO answers (+22% and +21%), while the third one had a lower improvement (+4%) probably due to his higher ICS score.

Parameters obtained during clinical performance tests indicate an improved involvement of the prosthetic leg while performing common daily tasks, which were performed at increased speed. For both 6MWT and TUG, a positive variation was observed, above the minimum detectable change in amputated individuals (MDC) of 45.0 m and 1.28s, respectively, as reported in literature.^27,28^

Certain biomechanical characteristics, like cadence, step width and all gait phase durations express as DoA are characterized by values that do not vary consistently or with a remarkable increment or decrement. Thus, the effect of the socket on these aspects of the gait can be considered minimal.

While presenting these main advantages over ICS sockets, this new hybrid sub-ischial design does not present any remarkably negative variation on typical gait parameters. Asymmetry between legs does not vary significantly and consistently, with non-negligible percentage variation typically associated with a higher standard deviation that does not allow to interpret the results obtained with different sockets as different. Improvements on gait velocity and stride length (around +10% or more for both measures and for each patient) with a negligible cadence variation (between +1% and +3%) could indicate a safer gait, with an improvement in walking speed mainly caused by longer footsteps.

Another interesting result is the change in the duration of double support phase within the gait cycle of the affected limb expressed as percentage of the whole cycle. In fact, it is shown how double support phase occupies a smaller amount of the gait cycle because of the increase in velocity.^29^ This can be considered as another sign of improvement in subjects’ walking, since increased values of double support duration in healthy subjects is typically found in the elderly.^30^

These results are in line with the those obtained with the NU-FlexSIV socket, where gait analysis parameters are unaffected by a lower brim, while hip RoMs are improved as expected by a design that does not contain the ischial ramus inside the prosthesis.^2^ New sub-ischial sockets represent a new possibility to improve the quality of life of the individuals affected by lower limb amputations. The material used for HySS, biomedical silicone, allows to precisely customize the morphology, thickness, stiffness and color to produce socket that combine desirable features.^31^ The results obtained from this case series should be considered preliminary due to the low number of participants involved. Future work will consist of a larger cohort of individuals with limb loss.

## CONCLUSION

The HySS can be considered an improvement over ischial containment sockets, because of the potential to overcome some common problems with ICS designs while achieving similar performance in other aspects of use.

## DECLARATION OF CONFLICTING INTERESTS

All the authors declare to have no conflicts of interest to declare.

## AUTHOR CONTRIBUTION

- **Roberto Pellegrini**: design and fabrication of HySS, supported the writing of the manuscript.
- **Gabriele Denza**: acquisition, analysis of the data, led the writing of the manuscript.
- **Stefano Brunelli**: conceived idea of the work, led the writing of the manuscript.
- **Daniele Zenardi**: design and fabrication of HySS, supported the writing of the manuscript.
- **Matteo Imperio**: acquisition, managed the data files, drafted the manuscript.
- **Giuseppe Vannozzi**: revised the manuscript critically for important intellectual content, supported the data analysis, interpretation of the data.
- **Marco Traballesi**: revised the manuscript critically for important intellectual content.

## SOURCES OF SUPPORT

The authors received no financial support for the research, authorship or publication of this article.

## ETHICAL APPROVAL

This case study was a pilot study; a forthcoming larger study is planned for which ethics committee approval is pending.
